# A Model of Electrically Stimulated Auditory Nerve Fiber Responses with Peripheral and Central Sites of Spike Generation

**DOI:** 10.1007/s10162-016-0608-2

**Published:** 2017-01-04

**Authors:** Suyash Narendra Joshi, Torsten Dau, Bastian Epp

**Affiliations:** Hearing Systems group, Technical University of Denmark, Ørsteds Plads Building 352, 2800 Kongens Lyngby, Denmark

**Keywords:** electrical stimulation, auditory nerve, cochlear implants, computational models, integrate-and-fire neuron

## Abstract

A computational model of cat auditory nerve fiber (ANF) responses to electrical stimulation is presented. The model assumes that (1) there exist at least two sites of spike generation along the ANF and (2) both an anodic (positive) and a cathodic (negative) charge in isolation can evoke a spike. A single ANF is modeled as a network of two exponential integrate-and-fire point-neuron models, referred to as peripheral and central axons of the ANF. The peripheral axon is excited by the cathodic charge, inhibited by the anodic charge, and exhibits longer spike latencies than the central axon; the central axon is excited by the anodic charge, inhibited by the cathodic charge, and exhibits shorter spike latencies than the peripheral axon. The model also includes subthreshold and suprathreshold adaptive feedback loops which continuously modify the membrane potential and can account for effects of facilitation, accommodation, refractoriness, and spike-rate adaptation in ANF. Although the model is parameterized using data for either single or paired pulse stimulation with monophasic rectangular pulses, it correctly predicts effects of various stimulus pulse shapes, stimulation pulse rates, and level on the neural response statistics. The model may serve as a framework to explore the effects of different stimulus parameters on psychophysical performance measured in cochlear implant listeners.

## **INTRODUCTION**

Patients with severe hearing loss or deafness are commonly prescribed with cochlear implants (CIs). The CIs bypass the impaired mechano-electrical transduction pathway through the cochlea and directly stimulate the auditory nerve fibers (ANFs) with electric pulses. CI signal processing strategies aim to mimic the cochlear processing of the acoustic inputs and to provide the CI listeners the “essential” cues for successful communication. Primarily, they extract the slowly-varying envelopes of the acoustic signals and stimulate the ANFs with a train of biphasic pulses modulated with the processed envelope (Wilson et al. 1991). Although most CI listeners can achieve some speech intelligibility in quiet with this strategy, they also face great difficulties in understanding speech in background noise and in other psychophysical tasks related to pitch and melody perception as well as sound localization (Wilson and Dorman 2008). Deficits in the temporal coding in the electrically stimulated ANFs may contribute to these perceptual difficulties of the CI listeners. Despite significant efforts in the development of signal processing strategies for better and efficient processing of the acoustical cues, the improvements in the performance of the CI listeners have been minimal and have been related mainly to the more advanced cue extraction strategies (e.g. advanced combination encoding, continuous interleaved sam-pling etc.) or to the current steering strategies that reduce the current spread in the cochlea (Bierer 2010). For the CI stimulation strategies to be further beneficial for the listener, the ANFs must be able to encode the envelope cues delivered by the CI. A better understanding of the stimulus-response relationship of the ANF for electrical stimulation seems thus crucial for the development of novel and more efficient stimulation strategies. Quantitative models, particularly those concerned with temporal aspects of ANF responses, can be a useful tool to characterize such a relationship.

Clinical CI devices use trains of symmetric biphasic pulses for the stimulation due to safety regulations. Contemporary accounts of electrical stimulation of the ANFs assume that the cathodic phase of the biphasic pulse depolarizes the neural membrane and generates a spike while the anodic phase hyperpolarizes the membrane and to balance the charge in the cochlea. However, results from several studies have shown that an anodic pulse can also generate a spike and that responses measured at the nerve trunk to the anodic phase exhibit shorter spike latencies than responses to the cathodic phase (van den Honert and Stypulkowski 1984; Miller et al. 1999; Shepherd and Javel 1999). Biophysical models that consider detailed cochlear morphology and its effects on the charge conduction through the cochlea show that an anodic pulse depolarizes the neuron at a location more distant from the stimulating electrode than a cathodic pulse (Rubinstein 1991; Rattay et al. 2001). These models suggest that the site of spike generation along the ANF differs for anodic and cathodic pulses. The site of spike generation is crucial since it determines the delay with which the spike arrives at further processing stages along the auditory pathway. A difference in spike latencies between the responses to anodic and the responses to cathodic pulses amounts to approximately 200 μs in cats (Miller et al. 1999) and possibly up to 400 μs in humans (Rattay et al. 2001, 2013; Undurraga et al. 2013). The effect of multiple sites of spike generation on spike latencies has also been reported in ANF responses to stimulation with biphasic pulses (van den Honert and Stypulkowski 1984; Miller et al. 1999; Shepherd and Javel 1999). Since robust temporal coding depends on the precision in spike timing, the site of spike initiation as well as the uncertainty related to it can affect the temporal coding in ANFs stimulated with symmetric biphasic pulses.

The ANF responses in cats show significantly lower thresholds for cathodic pulses than for anodic pulses (Miller et al. 1999). Therefore, it has been assumed that only the cathodic phase of a biphasic pulse will generate a spike. Based on this assumption, state-of-the-art quantitative models of ANF responses have mainly focused on the responsiveness of the ANF to the depolarizing cathodic phase (Bruce et al. 1999a; Hamacher 2004; Fredelake and Hohmann 2012; Goldwyn et al. 2012) or on inhibitory properties of the hyperpolarizing anodic phase (Rubinstein et al. 2001; Horne et al. 2016). Any charge-balanced pulse can be decomposed into anodic and cathodic charges, and responses to various pulse shapes are a consequence of the sensitivity to the single pulse phases and the interaction between these. Hence, the comparison of responses to different pulse shapes can be used to explore the response behavior of the electrically stimulated ANF. Current state-of-the-art models cannot account for the response statistics observed in the available data obtained with various pulse shapes (Joshi et al. 2014) and the effects of stimulation rate and level of pulse-train stimuli on the ANF responses (Bruce et al. 1999b; Goldwyn et al. 2010, 2012). Hence, these models cannot easily be generalized to assess the limitations and possible benefits of different CI stimulation strategies that aim to convey temporal information.

The current study presents and evaluates a computational model of the ANF responses for electrical stimulation. The model is based on the idea that there exist at least *two sites of excitation* along the ANF and that these sites differ in their *sensitivity to either cathodic or anodic charges*. Each unmyelinated node along the ANF can generate spikes in response to an extracellular voltage. However, for simplicity, the model presented in the current study considers all nodes along the peripheral axon of the ANF as one site of excitation and those along the central axon of the ANF as the other site of excitation. The model incorporates dynamic feedback loops that enable the prediction of response properties such as facilitation, accommodation, refractoriness, and spike-frequency adaptation. The model is evaluated for stimulus conditions with various pulse shapes and pulse trains of different stimulation rate and level.

## **THE MODEL**

### Structure

**FIG. 1:**
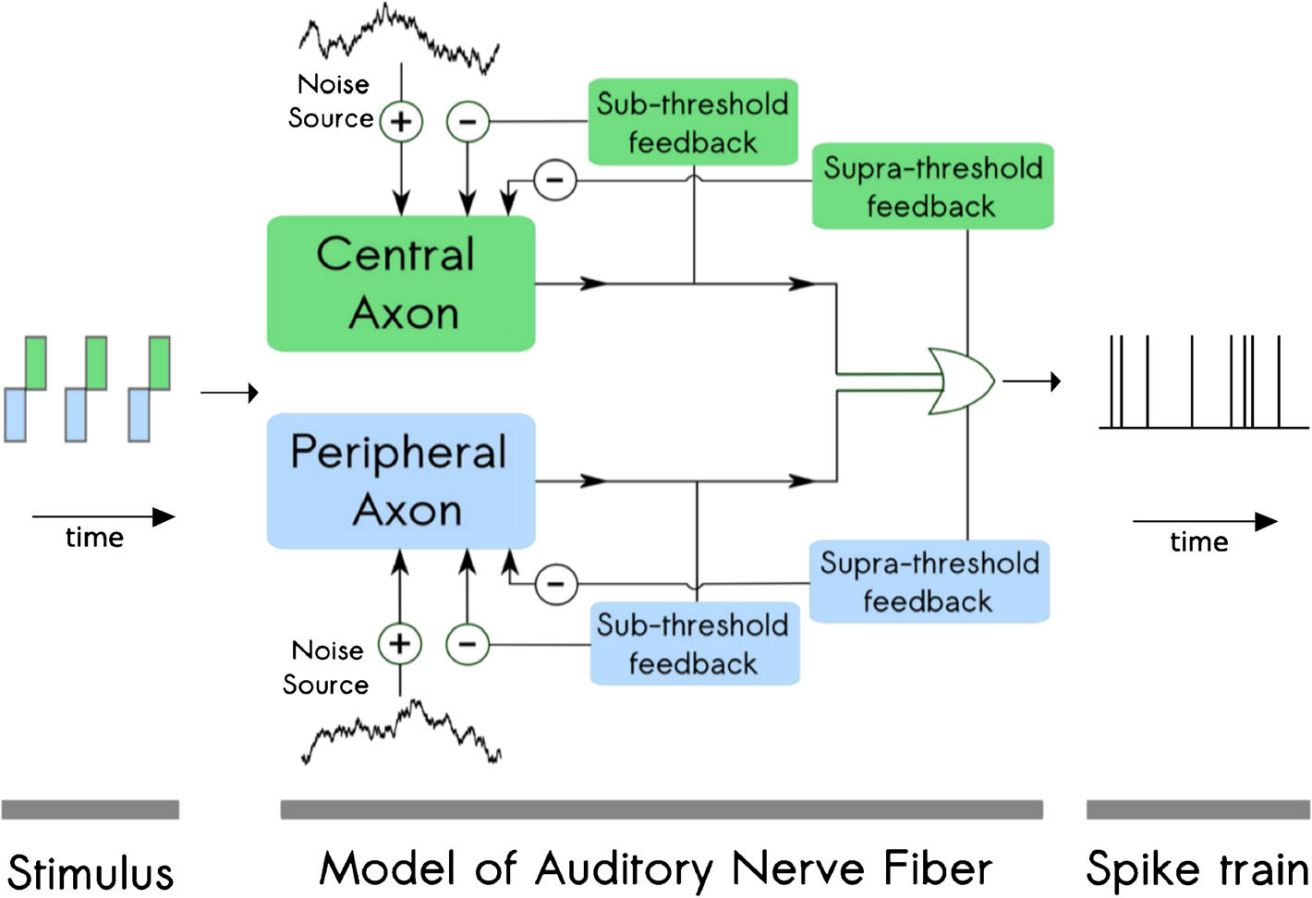
Structure of the proposed model. The ANF is modeled by a network of two point neurons describing the peripheral and the central axon of the ANF. The peripheral axon is excited by cathodic (negative) current and inhibited by anodic (positive) current. The central axon is excited by anodic current and inhibited by cathodic current. Independent noise inputs to each of the axons introduce stochasticity in the membrane potential. The two adaptive feedback currents account for subthreshold and suprathreshold adaptive properties observed in the ANF responses. The interaction between the two axons is modeled with an “OR” logic gate, selecting the first spike generated by either of the axons. The model receives the stimulus waveform as an input and provides spike times as the output.

The structure of the model is shown in Figure 1. A single ANF is divided into two parts: a peripheral axon and a central axon. The peripheral and central axons are each described by an exponential integrate-and-fire point neurons with two adaptive currents (Fourcaud-Trocmé et al. 2003; Brette and Gerstner 2005), which have been shown to account for the spike-time statistics of ANF responses (Rutherford et al. 2012). Based on the data from electrically stimulated ANFs in cats, the peripheral axon is assumed to be excited by cathodic charge and inhibited by anodic charge. The central axon is assumed to be excited by anodic charge and inhibited by cathodic charge (Miller et al. 1999). Subthreshold and suprathreshold adaptation are included in the model via two feedback loops which continuously modify the membrane potential. Finally, each axon includes an independent noise source to model the stochastic response properties of the ANF. In the model presented here, the two axons of the ANF are modeled in parallel. The differences in spike times between the spikes generated at the peripheral and the central axons are achieved by the differences in membrane characteristics.

Both model axons simultaneously calculate the membrane voltage *V* in response to the stimulus input *I*_*Stim*_:

1 $$ C\frac{dV}{dt}=h(V)-{I}_{sub}-{I}_{supra}+{I}_{\mathrm{Noise}}+{I}_{\mathrm{Stim}} $$

where *C* is the membrane capacitance, *I*_sub_ and *I*_supra_ are subthreshold and suprathreshold adaptation currents, and *I*_Noise_ is the noise current. *h*(*V*) describes the passive filtering of the stimulus along with the exponential upswing of the membrane potential during spike generation in the axon. The noise term *I*_Noise_ introduces stochasticity in the membrane voltage and is modeled as a random process with a Gaussian amplitude distribution and a power spectrum proportional to 1/*f* ^*α*^, inspired by the recordings of stochasticity in neural membrane voltages (Verveen and Derksen 1965). The exponent α determines the spectrum of the noise such that if α equals 0, the noise has a flat spectrum. If α is either 1 or 2, the noise spectrum decays with 3 or 6 dB per octave, respectively.

The passive filtering of the stimulus in each axon, *h*(*V*), is described as the sum of a linear and an exponential function:

2 $$ h(V)=-{g}_L\left(V-{E}_{\mathrm{L}}\right)+{g}_L\varDelta T\ {e}^{\left(\frac{V - {v}_{\mathrm{threshold}}}{\varDelta T}\right)} $$

where *g*_*L*_ is the membrane conductance, *E*_L_ is the resting membrane potential, ∆*T* represents the slope factor of the exponential function, and *v*_threshold_ is the threshold potential. In common integrate-and-fire models, a spike is indicated when the membrane potential crosses *v*_threshold_. In the exponential integrate-and-fire model described in Eq. 2, the membrane potential continues to increase after it has crossed *v*_threshold_. The increase of the membrane potential beyond *v*_threshold_ is exponential and the rate of the increase is determined by the slope factor ∆*T*. To indicate a spike discharge in this model, the peak voltage *v*_peak_ is introduced which indicates the membrane voltage at which the spike is generated. The corresponding time of spiking is denoted as *t*_spike_. After spiking, the membrane potential is reset to *v*_reset_.

The subthreshold and suprathreshold adaptive currents *I*_sub_ and *I*_supra_ are described in Eqs. 3 and 4, respectively:

3 $$ {\tau}_{\begin{array}{c}sub\\ {}\ \end{array}}\frac{d{I}_{sub}}{dt}={a}_{sub}\left(V-{E}_L\right)-{I}_{sub} $$

4 $$ {\tau}_{\begin{array}{c}\mathrm{supra}\\ {}\ \end{array}}\frac{d{I}_{\mathrm{supra}}}{dt}={a}_{\mathrm{supra}}\left(V-{E}_L\right)-{I}_{\mathrm{supra}} $$

with the conductances *a*_sub_ and *a*_supra_ and the time constants of the subthreshold and suprathreshold adaptation $$ {\tau}_{\begin{array}{c}sub\\ {}\ \end{array}} $$ and $$ {\tau}_{\begin{array}{c}\mathrm{supra}\\ {}\ \end{array}} $$, respectively. The magnitude of the adaptation currents depends on the membrane potential, *V*.

The interaction between the two axons is modeled with an “OR” logic gate that selects one output from the two inputs. This allows both axons to generate spikes independently, and the OR gate selects the axon that spikes first, indicated by *t*_spike_. The spike triggers an adaptation process that modifies the suprathreshold adaptation current, described in Eq. 4, by an offset *b*, which accounts for the spike-rate adaptation (Brette and Gerstner 2005). Irrespective of which axon generated a spike, the spike-triggered adaptation is applied to both the peripheral and the central axon. After a spike has occurred, the neuron is set into an absolute refractory period (ARP; Miller et al. 2001b). During this period, no spike can be generated irrespective of the level of the stimulus current. Unlike traditional integrate-and-fire models that describe the ARP as the *dead time*, here both axons continue to integrate the membrane potential during the ARP. However, during this period, the membrane only receives the input from the subthreshold and suprathreshold adaptation currents as well as the membrane noise, but no input from the stimulus.

The input to the model is a temporal waveform of the stimulus, *I*_stim_(*t*), in which *I* ^+^(*t*) is the anodic (positive) charge and *I* ^−^(*t*) is the cathodic (negative) charge. To allow the peripheral axon to be excited by a cathodic charge and inhibited by an anodic charge while using the same underlying equation, the input to the peripheral axon is the inverted temporal waveform of *I*_stim_(*t*). An additional parameter, *β*, is included in the model to vary the effect of the inhibitory phase on spike generation. When *β* equals 0, the inhibitory phase of the stimulus is fully removed and when *β* equals to 1, the inhibitory phase remains unchanged. As a result, the total input stimulus waveform to the two axons can be expressed as:

5 $$ {I}_{\mathrm{Stim}\ \left(\mathrm{central}\right)}(t)={\beta I}^{-}(t)+{I}^{+}(t) $$

6 $$ {I}_{\mathrm{Stim}\ \left(\mathrm{peripheral}\right)}(t)=-\left({I}^{-}(t)\ {+\beta I}^{+}(t)\right) $$

where *I*_Stim (peripheral)_ represents the stimulus input entering the peripheral axon, *I*_Stim (central)_ represents the stimulus input entering the central axon, *I* ^−^ is the cathodic charge, *I* ^+^ is the anodic charge and *β* is the scaling factor for the inhibitory charge. Both axons simultaneously receive the stimulus input and independently calculate the membrane potential at each location.

### Parameterization

The model parameters that determine the charge integration properties of the neural membrane (*g*_L_ and *C*) and stochasticity of neural responses (*I*_Noise_) were estimated from ANF responses to electrical stimulation with monophasic anodic and cathodic pulses in cats. The method used to estimate these parameters is described in the following paragraphs. The other remaining parameters of the model were estimated to best represent the responses to monophasic stimulation. The complete set of obtained values is presented in Table 1.

**TABLE 1:** Complete set of obtained values

	Peripheral neuron	Central neuron
Membrane conductance, *g*_L_	1.1 mS	2.7 mS
Membrane capacitance, *C*	856.96 nF	1772.4 nF
Slope factor, ∆*T*	10 mV	4 mV
Resting potential, *E*_L_	−80 mV	
Threshold potential, *v*_threshold_	−70 mV	
Peak potential, *v*_peak_	24 mV	
Reset potential, *v*_reset_	−84 mV	
Noise shaping parameter, *α*	0.8	
Inhibitory compression, *β*	0.75	
Subthreshold adaptation time constant, $$ {\tau}_{\begin{array}{c}sub\\ {}\ \end{array}} $$	250 μs	250 μs
Suprathreshold adaptation time constant, $$ {\tau}_{\begin{array}{c}\mathrm{supra}\\ {}\ \end{array}} $$	4500 μs	2500 μs
Subthreshold adaptation conductance, *a*_sub_	2 mS	
Suprathreshold adaptation conductance, *a*_supra_	3 mS	
Dead time	500 μs	

The parameters related to the charge integration properties of the integrate-and-fire models have been determined based on strength-duration data from neurons (e.g. Goldwyn et al. 2012). The strength-duration function represents the neural firing response threshold as a function of the pulse duration and can be described using the two parameters rheobase and chronaxie. Although previous studies described the strength-duration data by an exponential function (van den Honert and Stypulkowski 1984; Shepherd et al. 2001), it has been suggested that, for extracellular stimulation, a linear function describing the threshold as inversely related to the pulse duration provides a better description of the data (Nowak and Bullier 1998). In this case, the positive *y*-intercept of a regression line fit to the threshold data corresponds to the rheobase. The slope of the regression line divided by the rheobase corresponds to the chronaxie. This approach was used in the present study to derive the values for the rheobase and the chronaxie for monophasic anodic and cathodic pulses based on the ANF responses reported in Miller et al. (1999). The parameters *g*_L_ and *C* in Eqs. (1) and (2) were derived using these rheobase and chronaxie values.

Properties of the stochasticity of the neural responses have been described with a firing efficiency (FE) function which reflects the probability of spiking as a function of the stimulus level (Verveen 1961). The FE function generally has a sigmoidal shape and can be approximated by an integral of a Gaussian distribution with mean *θ* and standard deviation *σ*. The mean (*θ*) specifies the stimulus level that evokes a neural response with a probability of 0.5 and is defined as the threshold for ANF spiking. Sigma (*σ*) is a measure of the stochasticity of the neuron’s response to a stimulus at a given level. A normalized measure that combines *θ* and *σ*, the relative spread (RS), is obtained by taking the ratio of the standard deviation to the threshold of the neuron spiking (*σ*/*θ*). RS values are often reported to describe the normalized dynamic range of the ANFs (Miller et al. 1999). Here, the RS values reported by Miller et al. (1999) for anodic and cathodic pulses were used to determine the intensity of *I*_Noise_ entering the neural membrane by setting the variance of the underlying normal distribution to *σ* ^2^. The initial simulations showed that the spectral shape of *I*_Noise_, which changes with the parameter, affected the spiking threshold for monophasic stimulation. The value of *α* was chosen such that the predicted threshold for stimulation with monophasic pulses matched the data of Miller et al. (1999).

## **METHODS**

The effects of commonly used stimulus parameters, such as the pulse phase duration (PPD), interphase gap (IPG), stimulation pulse rate, and pulse level on the model responses were evaluated and compared to measured ANF responses. The response statistics from measured ANF responses were obtained by digitizing the individual data points from the corresponding studies. The stimulus conditions and the statistical measures to describe the neural responses were chosen to be identical with the respective studies in order to facilitate a comparison of the predictions with the data. The model was implemented in MATLAB (version 2016a, The MathWorks Inc., Natick, MA). The differential equations of the model were solved using the forward Euler method with an integration time step of 1 μs. All simulations were run with a constant set of parameters shown in Table 1.

### Stimuli

#### Single Pulses

**FIG. 2:**
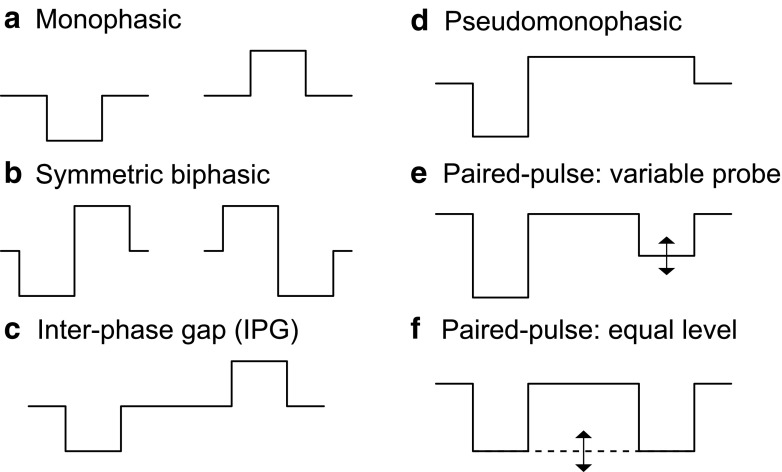
Stimulus conditions used to evaluate the model. **a** Monophasic cathodic and anodic pulses. **b** Symmetric biphasic pulses, with either cathodic or anodic leading polarity. **c** Symmetric biphasic pulse with an interphase gap (IPG). **d** Pseudomonophasic, charge-balanced pulse with different durations of the single pulse phases. **e** Paired pulse stimulus for the variable probe condition with constant conditioner (first) pulse and varied probe (second) pulse. **f** Paired pulse stimulus for the constant level condition, with conditioner and probe pulse at the same level.

The considered pulse configurations for single pulses are illustrated in Figure 2a–d. Although monophasic pulses of either polarity (Fig. 2a) can excite the ANFs, charge balancing is required for CI stimulation to avoid tissue damage. Symmetric biphasic pulses (Fig. 2b) can be regarded as the simplest charge balanced pulses, created by a monophasic pulse of one polarity immediately followed by the corresponding pulse of the opposite polarity. A drawback of the symmetric biphasic pulses is the neural interaction between the phases of opposite polarity (van den Honert and Mortimer 1979). Alternative pulse shapes have been used to reduce the interaction between the phases of opposite polarities in a symmetric biphasic pulse. One strategy is to introduce a silent gap between the phases of opposite polarities (Fig. 2c). If such an IPG is large enough, the effect of the second phase on the neural response is minimal. A second strategy to reduce the interaction between the two phases with opposite polarities is to use asymmetric pulse shapes which are characterized by different PPD of the two phases (Fig. 2d). For any given pulse duration and level of the leading phase, the second phase is made longer in order to neutralize the charge, but with a lower amplitude than for symmetric, biphasic pulses. The reduced amplitude of the second phase decreases its influence on the membrane potential. Since the duration of the two phases of opposite polarity is no longer the same and this pulse shape approximates monophasic stimulation, they are referred to as pseudomonophasic pulses.

In the present study, predictions were obtained for monophasic pulses with PPD of 25 to 500 μs of either anodic or cathodic polarity, and for biphasic pulses of either anodic or cathodic leading polarity with 25 μs to 10,000 μs PPD. The predicted response statistics for monophasic and biphasic pulses were compared with the data from Miller et al. (1999, 2001b), Rubinstein et al. (2001), Shepherd and Javel (1999), Smith and Finley (1997), and Bruce et al. (1999a). To test the effect of IPG in the model, thresholds were predicted for anodic leading and cathodic leading symmetric biphasic pulses with a PPD of 20 to 100 μs/phase and for IPGs from 2 to 200 μs. The predicted effects of IPG and PPD on the response statistics were compared to the data from Ramekers et al. (2014) and Shepherd and Javel (1999). Furthermore, predictions were obtained for pseudomonophasic pulses with a leading short cathodic phase of 40-μs duration, followed by an anodic pulse with durations between 40 and 5000 μs. The effect of the anodic phase duration on the response statistics was compared to data from Miller et al. (2001).

#### Paired Pulses

Any stimulus, irrespective of whether it generates a spike or not, affects the neural response to a subsequent stimulus. Such interaction effects can be measured by stimulation with two subsequent pulses. In such paired pulse stimuli, the first pulse is referred to as the “conditioner” and the second pulse is referred to as the “probe.” Two stimulus paradigms have been used to assess the effect of the conditioner on the probe. In one paradigm, the conditioner is presented at a fixed level and the probe threshold is measured for different delays of the probe (Fig. 2e). This condition is referred to as *the variable-probe condition*. In the second paradigm, the conditioner and the probe are presented at the same level and with different relative delays (Fig. 2f). This condition is referred to as *the constant-level condition*. By changing the level of the conditioner to be either below or above threshold, paired pulse stimulation has been used to characterize the effects of facilitation, accommodation, and refractoriness in the electrically stimulated ANFs.

The *subthreshold* conditioner refers to a pulse of low amplitude that does not generate a spike. In the variable-probe condition, the conditioner level was chosen to be either −0.9 or −2 dB below the threshold for a single pulse and probe thresholds were predicted for monophasic cathodic pulses of 100 μs PPD and inter-pulse delays ranging from 100 to 5000 μs. In the constant-level condition, the summation thresholds, i.e., the threshold for two pulses of equal level, were predicted for monophasic pulses of anodic or cathodic polarity for a PPD of 50 μs using interpulse delays in the range from 100 to 300 μs. A summation time constant was obtained by fitting the summation thresholds with an exponential function, as suggested by Cartee et al. (2006). The effects of subthreshold paired pulse stimulation in the model were compared to the data from Dynes (1996) and Cartee et al. (2006).

The *suprathreshold* conditioner refers to a pulse of higher amplitude that always generates a spike. In the variable-probe condition, the conditioner level was fixed at either +1, +2, +4, or +6 dB above the threshold level for a single pulse and thresholds were predicted for 100-μs-long monophasic cathodic probe pulses and interpulse delays ranging from 600 to 14,000 μs. In the constant-level condition, the probability of spiking for the second pulse was predicted for various interpulse delays, using pseudomonophasic pulses. The pseudomonophasic pulses were composed of either anodic or cathodic leading phase (40 μs) and a long-duration second phase (160 μs) of opposite polarity. In each condition, the conditioner and the probe were identical in their pulse shape. The conditioner and probe levels were kept constant at either +1 or +3 dB above the threshold for a single pulse and the interpulse delays were varied from 500 to 16,000 μs. The effects of the suprathreshold paired pulse stimulation were compared to the data from Dynes (1996) and Matsuoka et al. (2000).

#### Pulse Trains

Besides the pulse shape, pulse rate and stimulus level also affect the neural responses. Predictions were obtained for stimulation with pulse trains with rates from 200 to 10,000 pulses per second (pps). The pulse trains were 300 ms in duration and composed of symmetric biphasic pulses of either anodic or cathodic leading polarity. The amplitudes of the pulses in a pulse train were constant and varied across conditions between −10 and +10 dB above the threshold for a single pulse. The predictions were compared to the data from Javel (1990), Bruce et al. (1999b), Zhang et al. (2007), and Miller et al. (2008).

### Response Statistics

For single and paired pulses, the response statistics used to characterize the neural response included threshold, dynamic range, spike latency, and jitter. First, the spike probabilities were obtained by repeating the simulations 1000 times at multiple current levels. The probabilities were fit with an integrated Gaussian function described with mean *θ* and standard deviation *σ* to obtain the FE function. The threshold was defined as the value of *θ* of the FE function. The dynamic range of the neuron was estimated by calculating the RS as *σ*/*θ*. The spike latency distribution was calculated from the time delays of the generated spikes relative to the stimulus onset. The standard deviation of the spike latency distribution was then defined as the spike jitter.

For pulse trains, the response can be described by the spike rate and its variability, the dynamic range, the interspike interval (ISI) histogram, and the vector strength (VS). The spike rate is defined as the observed number of spikes per second for any given stimulus at a particular stimulus level. The variability in spike rate can be quantified using the Fano factor. The Fano factor is a normalized measure of the variance in spike rate and can be described as the ratio of the standard deviation of the spike rate to the mean spike rate, evaluated across repetitions of the stimulus. The dynamic range of the neuron was derived by calculating the rate-level function via simulations of the spike rate at multiple levels. The ISI histogram was considered to represent information related to the temporal dispersion of the spike times and was constructed with a bin-width of 1 ms. The VS was calculated to quantify the periodicity in the neural responses. It represents the phase locking of the spike times in response to sinusoidal stimulation:

7 $$ VS=\frac{1}{N}\sqrt{{{\left[\sum_{i=1}^N\mathit{\cos}\left(\frac{2\pi {t}_i}{T}\right)\right]}^2+\left[\sum_{i=1}^N\mathit{\sin}\left(\frac{2\pi {t}_i}{T}\right)\right]}^2} $$

where *N* is the total number of spikes used in the analysis, *t*_*i*_ is an individual spike time (in seconds), and *T* is a period of the sinusoid for which the synchronization of spike times is being assessed. The value of the VS can maximally be 1, indicating perfect phase locking, and minimally 0, indicating no phase locking to the stimulus frequency. In order to avoid any effect of the onset responses to the stimuli, the spikes appearing in the first 50 ms of the stimulus were excluded from the calculation of the VS. The effect of the pulse rate on the VS was obtained for the pulse train stimuli presented at a level of 1 dB above the threshold for a single pulse. The effect of level on the model responses was quantified by selecting stimulus levels which resulted in spikes rates from 4 to 500 spikes/s.

Changes in neural responses across time were investigated by constructing the peri-stimulus time histograms (PSTH) and wide-bin adaptive peri-stimulus time histograms (aPSTH) in response to pulse trains. The PSTH were constructed with a bin-width of 1 ms from responses to the pulse train stimuli presented at a level of 1 dB above the threshold for a single pulse. The aPSTH refers to the PSTH constructed with increasing time windows across the stimulus duration. Following Zhang et al. (2007), the aPSTH were constructed for temporal windows of 0–4, 4–12, 12–24, 24–48, 48–100, 100–200, and 200–300 ms from the time onset in response to a 300-ms-long pulse train.

## **RESULTS**

### Responses to Single-Pulse Stimulation

**FIG. 3:**
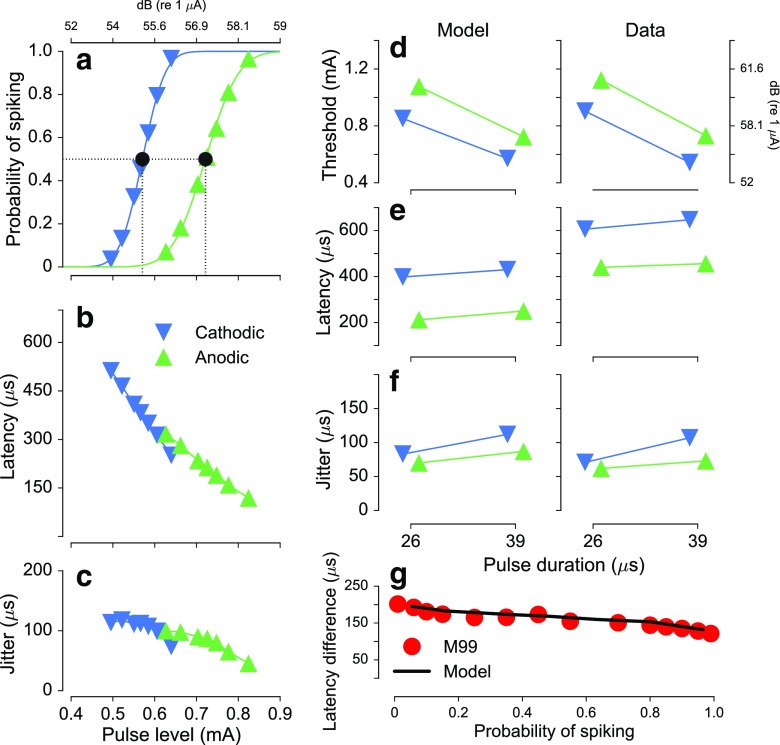
Responses to monophasic anodic (green downward pointing triangles) and cathodic (blue upward pointing triangles) pulses. **a** FE curves predicted by the model in response to 39-μs duration pulses together with a fit using an integrated Gaussian (*lines*). **b**, **c** Corresponding spike latency and jitter as a function of the pulse level. **d**–**f** Comparison of predicted thresholds, spike latencies, and jitter (*left*) with the corresponding data from Miller et al. (1999) (*right*) in response to monophasic pulses of 26- and 39-μs duration. **g** Predicted (*black line*) differences in spike latencies between cathodic and anodic monophasic pulses together with data from Miller et al. (1999) (*red circles*).

The simulated response statistics for *monophasic* anodic and cathodic pulses of 26- and 39-μs duration are shown in Figure 3. The effects of current level on the FE, the spike latency, and the jitter for anodic (green upward pointing triangles) and cathodic (blue downward pointing triangles) pulses of 39-μs duration are shown in Figure 3a–c, respectively. The responses to anodic and cathodic pulses are probabilistic, and the probability of spiking increases with an increase in stimulus level (Fig. 3a). The solid lines represent the FE functions fitted to the predicted probabilities with the 50 % points indicated as thresholds. The model predicts lower thresholds for cathodic than for anodic pulses. The spike latencies (Fig. 3b) are generally higher for cathodic than for anodic pulses and decrease with increasing pulse level. The jitter (Fig. 3c) decreases with increasing pulse level for both cathodic and anodic pulses.

The comparison of the predictions to data for 26- and 39-μs-long anodic and cathodic monophasic pulses is shown in Figure 3d–f. Thresholds (Fig. 3d) are higher for 26-μs duration than for 39 μs for both anodic and cathodic pulses. However, the pulse duration has only a minor effect on the spike latency (Fig. 3e) or its jitter (Fig. 3f). The predictions (left panels) and the data (right panels) are in good agreement, except for the absolute values of the spike latencies which are about 200 μs lower than the corresponding values in the data. Figure 3g shows the differences in spike latencies between anodic and cathodic pulses of 39 μs as a function of the probability of spiking. The predictions are indicated by the black line. For comparison, the data by Miller et al. (1999) are represented by the red circles. The difference between spike latencies for anodic vs cathodic pulses is about 200 μs for low spiking probabilities and reduces to about 150 μs for high spiking probability. The predictions are consistent with the data, i.e., the model can successfully account for the spike-latency differences between anodic and cathodic pulses.

**FIG. 4:**
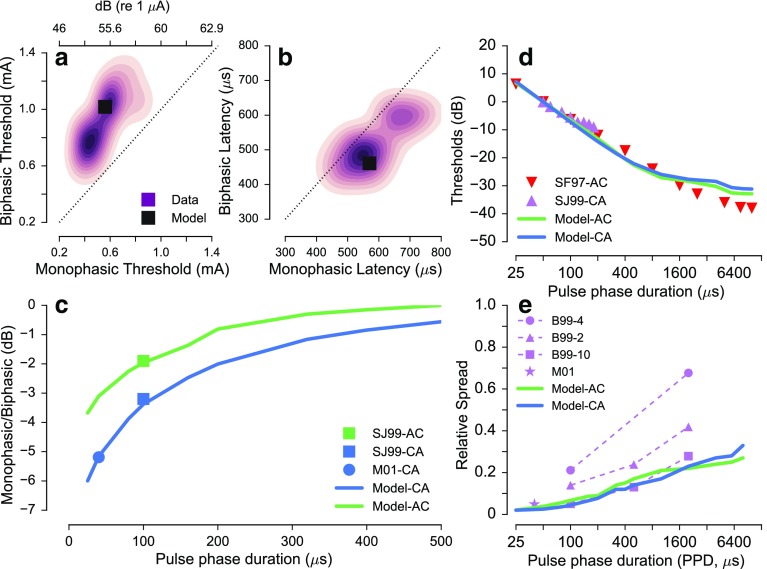
Responses to symmetric biphasic pulses. **a**, **b** Comparison of thresholds and spike latencies for a monophasic cathodic pulse and a cathodic leading symmetric biphasic pulse. Predictions are indicated by the *black squares*, and distributions of the data from Miller et al. (2001a) are shown by the purple kernel density functions. **c** Predicted effect of PPD on the difference between monophasic and biphasic pulses of both polarities (*lines*) along with corresponding data from Miller et al. (2001) (M01-CA) and Shepherd and Javel (1999) (SJ99AC, SJ99CA). **d** Predicted effect of PPD on the threshold for symmetric biphasic pulses of anodic (AC) or cathodic (CA) leading polarities (*lines*) along with the corresponding data from Shepherd and Javel (1999) (SJ99-CA) and Smith and Finley (1997; SF97-AC). **e** Predicted effect of PPD on the RS (*lines*) along with the corresponding data from Bruce et al. (1999) (B99-4, B99-2, B99-10) and Miller et al. (2001) (M01).

Figure 4 shows the simulated response statistics for *symmetric biphasic pulses*. Figure 4a represents the thresholds for cathodic leading biphasic pulses (ordinate) in comparison to those for cathodic monophasic pulses (abscissa). Figure 4b represents the corresponding results for the spike latencies. The black squares in Figure 4a, b represent the predictions. The shaded area indicates the kernel density function (estimated using function “KDEplot” of the seaborn toolbox in python) of the ANF responses in Miller et al. (2001a), whereby each shade represents a step of ten percentiles in the data. Both predictions and data show that the thresholds for biphasic pulses are higher than those for monophasic pulses (Fig. 4a) and that the spike latencies for biphasic pulses are lower than for monophasic pulses (Fig. 4b). Figure 4c shows the predicted differences in thresholds between monophasic and biphasic pulses with corresponding leading polarity (leading cathodic (CA); leading anodic (AC); shown as lines) as a function of PPD. The predictions show that the difference between monophasic and biphasic pulses is largest for smaller PPD and reduces with increasing PPD. In general, the difference in threshold between monophasic and biphasic pulses is lower for monophasic anodic and AC pulses than for monophasic cathodic and CA pulses. The predictions are consistent with the available data obtained for individual values of PPD (Miller et al., 2001, M01-CA with a circle; Shepherd and Javel 1999, SJ99 with squares).

Figure 4d, e shows the effect of PPD of symmetric biphasic pulses on threshold and RS. The predicted thresholds for CA pulses (indicated by the blue line) and AC pulses (indicated by the green line) are shown as a function of PPD. The thresholds have been normalized relative to the threshold for a biphasic pulse with 50 μs PPD. The thresholds are highest for short PPDs and decrease monotonically with increasing PPD, both for CA and AC pulses. This decrease is consistent with the data in Smith and Finley (1997) and Shepherd and Javel (1999), indicated by the downward (SF97) and upward pointing triangles (SJ99), respectively. The largest difference between the predictions and the data appears at longer PPDs beyond 2000 μs/phase, where the predictions are higher than the data observed by Smith and Finley (1997). In Figure 4e, the predicted RS values (solid lines) increase monotonically with increasing PPD, both for CA and AC pulses. This prediction is consistent with the trends in the data in Bruce et al. (1999) that also showed that the RS of an electrically stimulated ANFs in cat increased systematically with increasing PPD (B99-2, B99-4, and B99-10, green points in Fig. 4e). The absolute values of the predicted RS are consistent with the fiber labeled as B99-10 (indicated by purple square), while the remaining two fibers (B99-2, B99-4) show higher values of the RS.

**FIG. 5:**
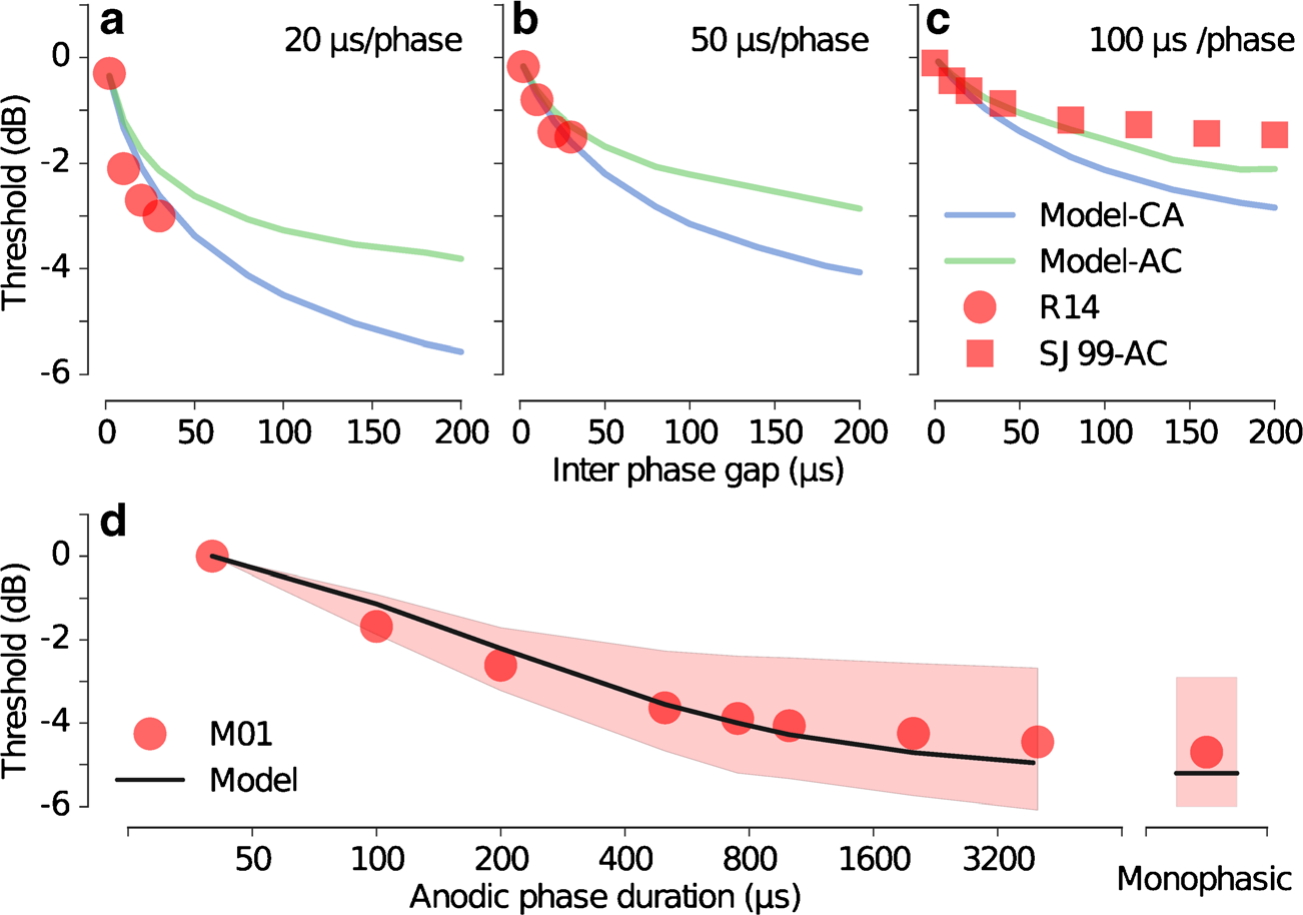
Effect of IPG on threshold for biphasic pulses of PPDs for (**a**) 20 μs/phase (**b**) 50 μs/phase, and (**c**) 100 μs/phase. Predictions (*lines*) are compared to the corresponding data from Ramekers et al. (2014) (R14) and Shepherd and Javel (1999) (SJ99-AC). **d** Thresholds for pseudomonophasic pulses as a function of the anodic phase durations along with the threshold for monophasic cathodic pulse for comparison. Predictions (*line*) are compared to the corresponding data from Miller et al. (2001a) (M01). The *shaded area* represents the spread of the data.

Figure 5 shows simulated response statistics and corresponding data for stimulation with symmetric and asymmetric pulse shapes. Figure 5a–c shows the effect of IPG on thresholds for biphasic pulses with PPDs of 20, 50, and 100 μs/phase. The predictions for AC and CA pulse shapes are shown as green and blue lines, respectively. The data from Ramekers et al. (2014) for 20 and 50 μs/phase are indicated by the red circles in Figure 5a, b, and those from Shepherd and Javel (1999) for 100 μs/phase indicated by the red squares in Figure 5c. The data of Ramekers et al. (2014) represented the average of the thresholds for CA and AC pulse shapes. The data of Shepherd and Javel (1999) indicate the thresholds for AC pulses. The thresholds decrease exponentially with increasing IPG both in the predictions and the data. The model also predicts an effect of PPD on the threshold decrease due to an IPG; the effect of IPG is higher for smaller PPDs (Fig. 5a) than for longer PPDs (Fig. 5c), and the effect of IPG is higher for CA pulses than for AC pulses. The predicted effect of PPD on the threshold reduction with increasing IPG is consistent with the corresponding data.

Figure 5d shows the predicted thresholds for *pseudomonophasic pulses* as a function of the duration of the second, anodic phase (black line). The thresholds were normalized relative to the threshold for a symmetric biphasic pulse with 40 μs PPD. The threshold for a cathodic monophasic pulse of 40 μs is also shown on the right for comparison. The corresponding mean data from Miller et al. (2001) are indicated by the red circles, and standard deviation of the data is represented by the shaded area. Both predictions and data show that the thresholds decrease monotonically with increasing anodic phase duration. Thresholds continue to decrease beyond 1000 μs asymptotically approaching the threshold for a monophasic cathodic pulse. The predictions are in good agreement with the data.

### Responses to Paired Pulse Stimulation

**FIG. 6:**
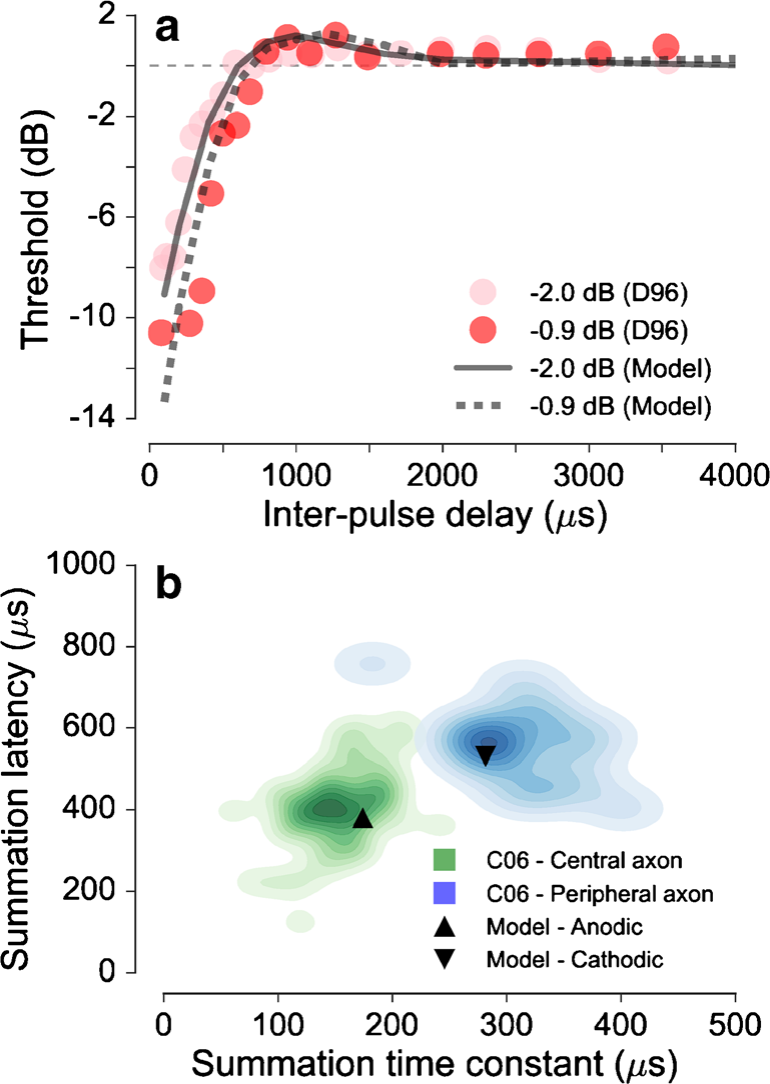
Responses to subthreshold paired pulse stimulation. **a** Predicted effect of conditioner level on the probe threshold as a function of the interpulse delays (*lines*) along with the corresponding data from Dynes (1996) (D96). **b** Comparison of the summation latencies obtained with the equal-level condition for the anodic and the cathodic pulses (abscissa) with the corresponding summation latencies (ordinate). Predictions (*triangles*) are compared with the corresponding distributions of the data of Cartee et al. (2006) (blue and green kernel density functions).

The simulated response statistics for paired pulses with a *subthreshold conditioner* are shown in Figure 6. Figure 6a shows the predicted thresholds (solid and dotted black lines) for paired pulses in the variable-probe condition as a function of the interpulse delay. The thresholds were normalized relative to the threshold for a single pulse, which is indicated by a dashed horizontal line. The predictions show that for interpulse delays below about 800 μs, the thresholds for the paired pulses are notably lower than the thresholds for a single pulse. This process of temporal integration of the charge has been referred to as facilitation. For interpulse delays above 800 μs, thresholds are elevated relative to the threshold for a single pulse. This form of subthreshold masking has been referred to as accommodation. After about 2000 μs, the threshold approaches the threshold for a single pulse. The amount of facilitation observed at delays below 800 μs depends on the level of the conditioner pulse. In the predictions, the effect of facilitation is slightly stronger for a −0.9 dB (dashed black line) subthreshold conditioner than for a −2.0 dB (solid black line) subthreshold conditioner. The predictions are consistent with the data of Dynes (1996) indicated by the light red circles (for the conditioner level of −2.0 dB) and in red circles (for the conditioner level of −0.9 dB).

In Figure 6b, predicted spike latencies (ordinate) and summation time constants (abscissa) are shown for paired-pulses stimulation in the equal-level condition. The black upward pointing triangle shows the results for anodic pulses, and the black downward pointing triangle shows the results for cathodic pulses. The summation time constant in the model is about 175 μs for the anodic paired pulses and 280 μs for the cathodic paired pulses. The summation latency amounts to 390 μs for the anodic pulses and 520 μs for the cathodic pulses. These predictions are well within the range of the data from Cartee et al. (2006) which are indicated by the kernel densities of the distribution of ANF responses for the peripheral site of spike generation (blue area) and the central sites of spike generation (green area). The color shades of the kernel density functions represent a step of ten percentiles of the data.

**FIG. 7:**
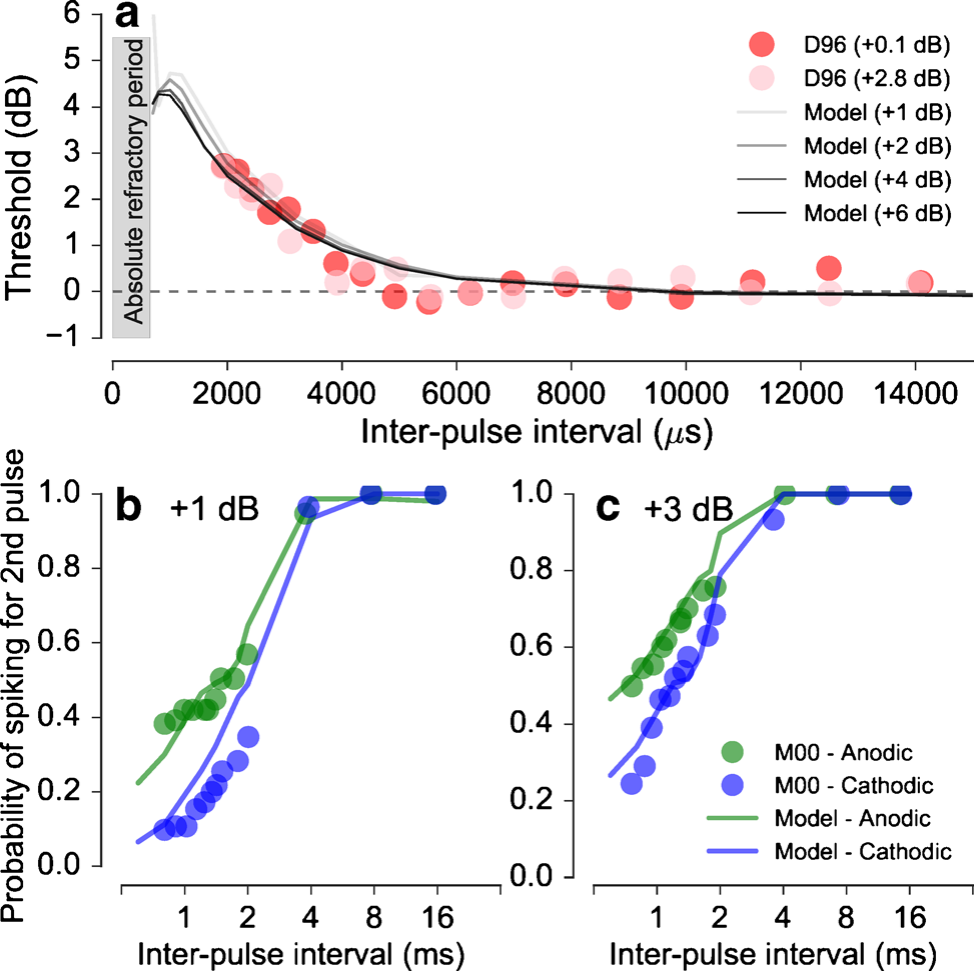
Responses to suprathreshold paired pulse stimulation. **a** Predicted effect of the suprathreshold conditioner level on the probe threshold as a function of the interpulse interval (*lines*) along with the corresponding data from Dynes (1996) (D96). **b**, **c** Probability of spiking in response to the probe measured in the equal-level condition for two suprathreshold levels +1 dB (**b**) and +3 dB (**c**). Predictions (*lines*) are compared to the corresponding data from Matsuoka et al. (2000) (M00). Note that single-pulse threshold for the cathodic pseudomonophasic pulse is lower (810 μA) than for the anodic pseudomonophasic pulse (885 μA).

The predictions for paired pulse stimulation with pseudomonophasic pulses with a *suprathreshold conditioner* are shown in Figure 7. Figure 7a shows the predicted thresholds for the paired pulses in the variable-probe condition as a function of the interpulse interval. The different lines indicate the results for different conditioner levels. The thresholds have been normalized relative to the threshold for a single pulse, indicated by the dashed horizontal line. The predictions show that there is no spike generation in the interval immediately following a spike produced by the suprathreshold conditioner, as indicated by the ARP of about 600 μs (shaded gray area). Previous studies have reported mean ARP within a range of 300 to 700 μs (Dynes 1996; Miller et al. 2001b; Imennov and Rubinstein 2009). Beyond the ARP, the thresholds are increased relative to the thresholds for a single pulse for interpulse intervals of up to about 5000 μs. This period where the threshold is elevated represents the refractory period of the neuron. The predictions show only a negligible effect of the conditioner level on the refractory period, and the thresholds approach the baseline level of a single pulse threshold at about 5000 μs. The predictions are in good agreement with data from Dynes (1996) that are indicated by the filled red and pink circles for the conditioner levels of +0.1 and +2.8 dB, respectively, as well as the estimates of Miller et al. (2001b) of the refractory period.

Figure 7b shows the probability of spiking for the second pulse in the case of suprathreshold paired pulse stimulation in the equal-level condition for a level of +1 dB re single pulse threshold. The results for a level of +3 dB are shown in Figure 7c. The blue and green lines indicate the results for cathodic and anodic pulses, respectively. The results show that the probability of spiking increases with increasing interpulse interval. The probability approaches 1 for interpulse intervals of about 5 ms, at both stimulus levels. For interpulse intervals below 5 ms, the probabilities are lower for cathodic than for anodic pulses. The difference between the probabilities for anodic and cathodic pulses decreases with increasing interpulse delays. The predictions are consistent with corresponding data from Matsuoka et al. (2000) represented as blue and green circles for cathodic and anodic pulses, respectively.

### Responses to Pulse Trains

**FIG. 8:**
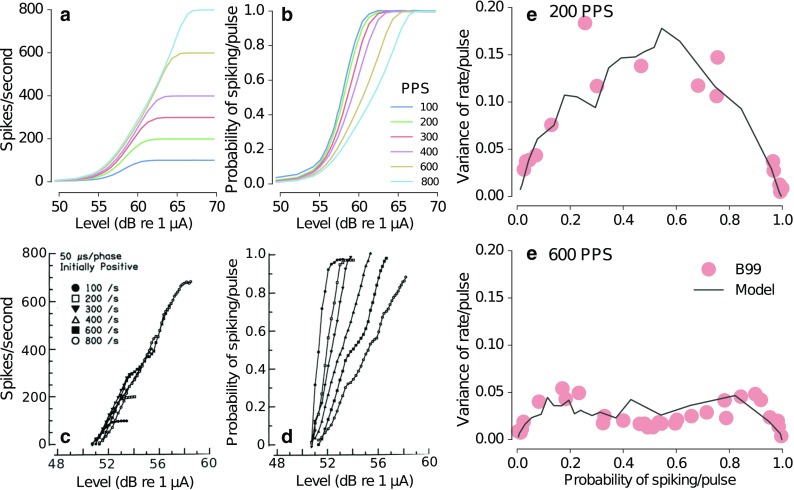
Predicted rate-level functions and spike rate variability in response to pulse train stimuli. **a** Predicted rate-level functions for stimulation rates ranging from 100 to 800 pps. **b** Predicted rate-level functions normalized to the stimulus pulse rate. **c** Corresponding measured rate-level functions for stimulation rates ranging from 100 to 800 pps from Javel (1990). **d** Normalized rate-level functions from Javel (1990). **e** Predicted variance in spike rate for stimulation with a pulse train of 200 pps (*line*) along with the data from Bruce et al. (1999b) (B99, *circles*). **f** Predicted variance for stimulation with a pulse train of 600 pps (*line*) along with the corresponding data from Bruce et al. (1999b) (B99, *circles*). The data in c and d have been reprinted from Javel (1990; Fig. 17.21) with kind permission of Springer Science + Business Media.

The predictions for pulse train stimulation are shown in Figure 8. The predicted rate-level functions (Fig. 8a) are indicated as spikes/s for pulse rates in the range from 100 to 800 pps. The predictions show a monotonic increase in spike rate with increasing level. For the corresponding pulse rates, the spike rates saturate at levels between 60 and 65 dB to spike rates equal to the pulse rate. The dynamic range of the neural response for pulse train stimuli can be extracted as a difference between the neuron’s threshold level and the level at which the spike rate saturates. To measure the effect of the pulse rate on the dynamic range of the neuron, the rate-level functions can be normalized by dividing the spike rate by the stimulus pulse rate. Figure 8b shows the corresponding normalized rate-level functions representing the probability of spike/pulse for pulse rates in the range from 100 to 800 pps. It can be seen that the dynamic range of the neuron is larger for higher pulse rates than for lower pulse rates. Despite some differences in the absolute stimulus levels, the predicted rate-level functions for the different pulse rates (Fig. 8a, b) are consistent with corresponding data shown in Javel (1990, Fig. 8c, d).

Figure 8e shows the variability in spike rate across the dynamic range of the neuron for the pulse rate of 200 pps. The normalized dynamic range (abscissa) is indicated as the probability of a spike/pulse. The predictions, indicated by the black lines, show that the variability of the response for 200 pps is largest for probabilities around 0.5. For 600 pps (Fig. 8f), the variability is largest at about 0.25 and 0.75 and slightly lower at intermediate probabilities around 0.5. The absolute variance in spike rate is larger for 200 pps than for 600 pps. The data reported in Bruce et al. (1999b), indicated by the red circles, show a comparable effect of the simulation rate on the variability of the response.

**FIG. 9:**
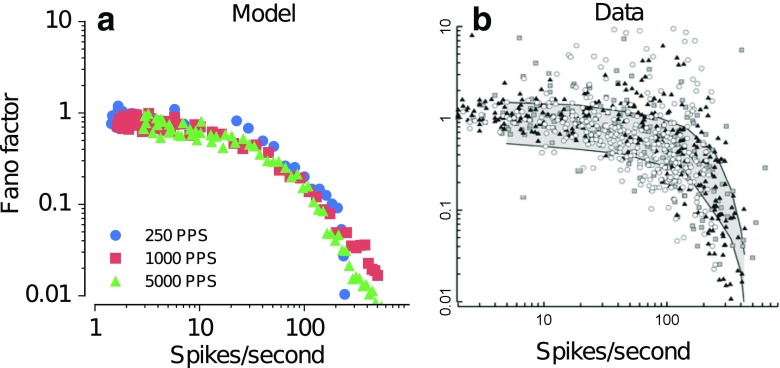
Fano factor derived from the predicted (**a**) and measured (**b**) ANF responses for stimulation with pulse trains of 250 pps (*circles*), 1000 pps (*squares*), and 5000 pps (*triangles*) at various stimulus levels as a function of the spike rate. The corresponding data (**b**) have been reprinted with kind permission of the Journal of the Association for Research in Otolaryngology, Springer Science + Business Media: Fig. 9 from Miller et al. (2008), © Association for Research in Otolaryngology 2007.

Figure 9a shows the predicted variability of spike rate in terms of the Fano factor as a function of the mean spike rate for stimulus pulse rates of 250 (blue), 1000 (red), and 5000 pps (green). Figure 9b shows the data of Miller et al. (2008) indicated as the Fano factor as a function of the mean spike rate for stimulus pulse rates of 250 (circles), 1000 (squares), and 5000 pps (triangles). The predictions (Fig. 9a) as well as the data (Fig. 9b) indicate that the Fano factor is larger for low spike rates (corresponding to low stimulus levels) and decreases with increasing spike rates (corresponding to increasing stimulus levels). The stimulation pulse rate shows only a marginal effect on the Fano factor. The predictions are roughly consistent with the data in Figure 9b. However, many data points, with each point corresponding to a different ANF, show notably higher values of the Fano factor (>1) than the predicted values, particularly at spike rates around 100 spikes/s, i.e., the high variability in the data across ANFs cannot be accounted for by the model.

**FIG. 10:**
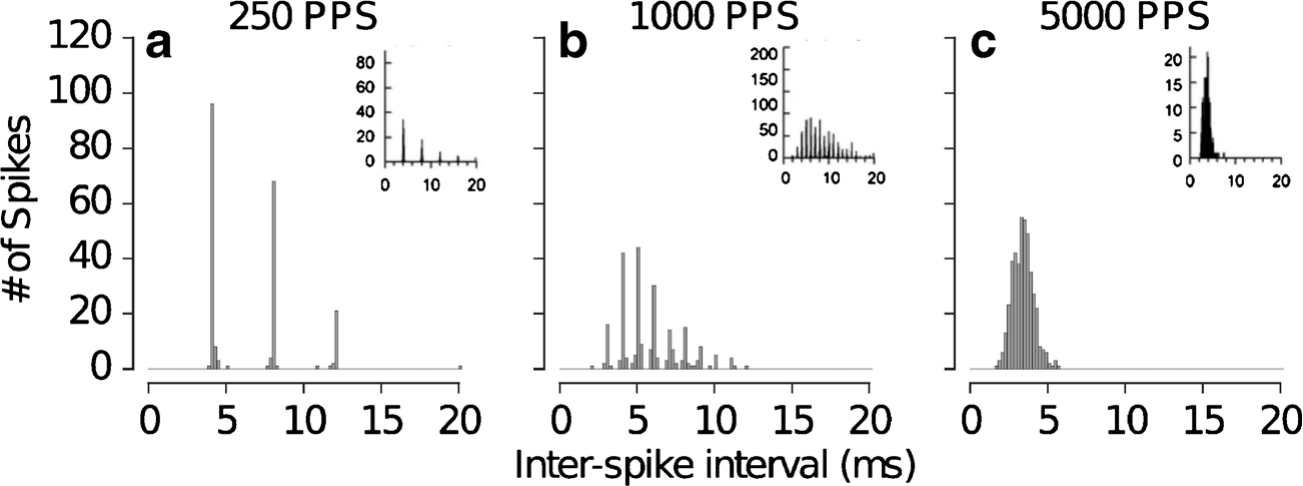
ISI histograms with a bin width of 1 ms derived from the predicted responses for stimulation with pulse trains of 250 pps (**a**), 1000 pps (**b**), and 5000 pps (**c**), along with the corresponding data from Miller et al. (2008) in the *insets*. The *data in the insets* have been reprinted with kind permission of the Journal of the Association for Research in Otolaryngology, Springer Science + Business Media: Fig. 1 from Miller et al. (2008), © Association for Research in Otolaryngology 2007.

**FIG. 11:**
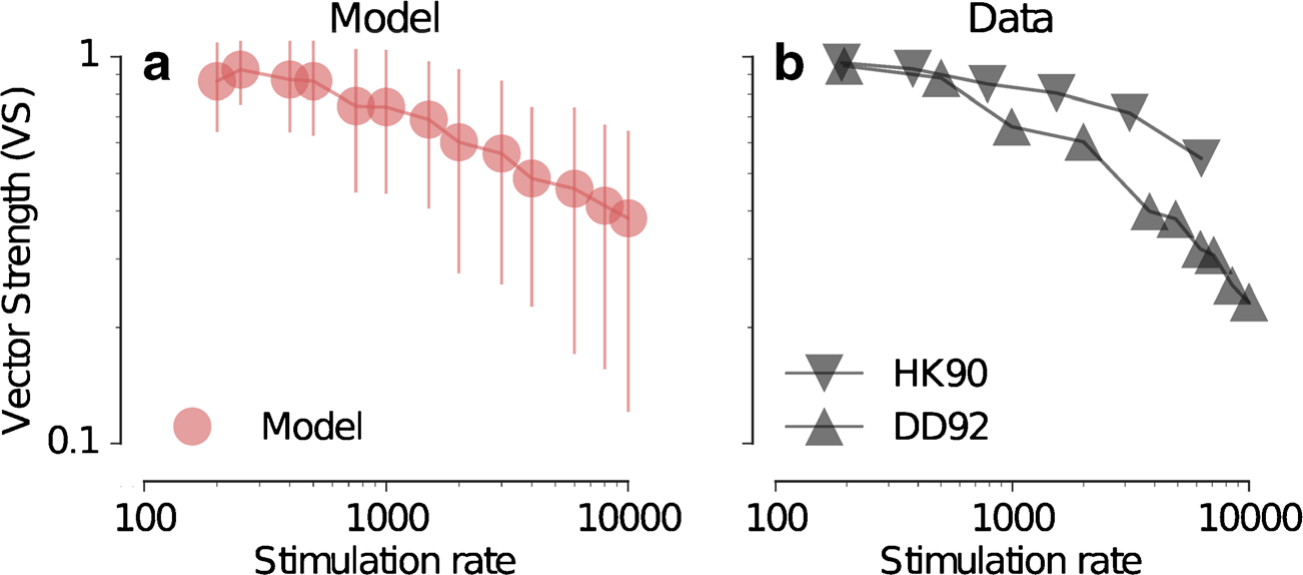
**a** Vector strength (VS) derived from the predicted responses as a function of the stimulation rate (pps). The *error bars* represent the standard deviation of the VS across 100 repetitions. **b** Corresponding data from Hartmann and Klinke (1990) (HK90, *downward pointing triangles*) and Dynes and Delgutte (1992) (DD92, *upward pointing triangles*).

**FIG. 12:**
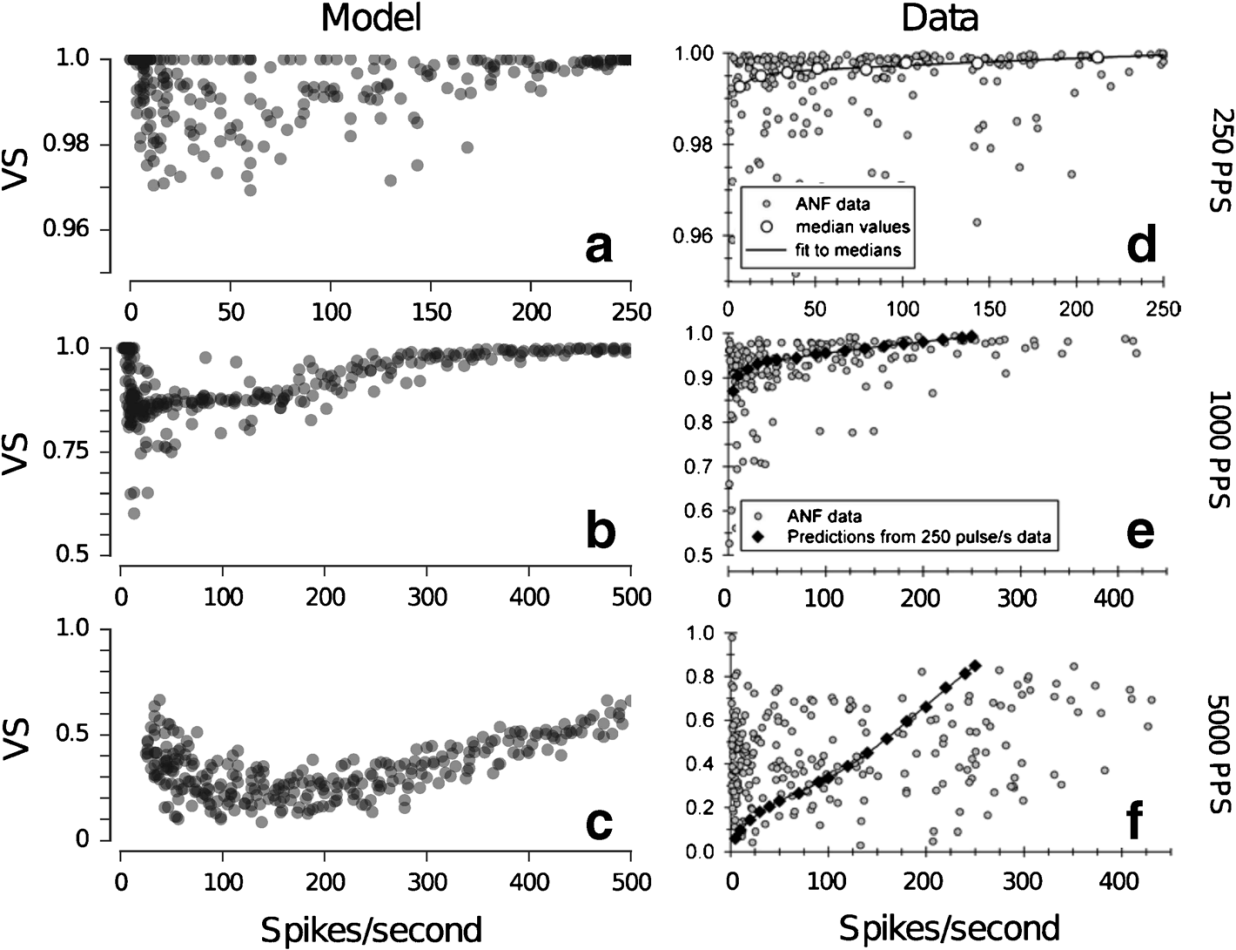
Predicted changes in VS as a function of the spike rate for stimulation with pulse trains of 250 pps (**a**), 1000 pps (**b**), and 5000 pps (**c**) along with the corresponding data from Miller et al. (2008) (**d**–**f**). The data have been reprinted with kind permission of the Journal of the Association for Research in Otolaryngology, Springer Science + Business Media: Fig. 8 from Miller et al. (2008), © Association for Research in Otolaryngology 2007.

Figures 10, 11, and 12 show the predicted response statistics related to the measures of temporal dispersion in the spike times, such as ISI histograms and vector strength (VS). Figure 10 shows the simulated ISI histograms for stimulus pulse rates of 250, 1000, and 5000 pps. The data from Miller et al. (2008) are indicated in the insets. For pulse trains with a rate of 250 pps (Fig. 10a), the histogram shows the largest peak at about 4 ms, which corresponds to the pulse train period representing the (inverse of the stimulation pulse rates). The secondary peaks in the histogram appear at multiples of 4 ms. A similar trend was found for pulse rates of 1000 pps (Fig. 10b) where the histogram shows multiple peaks in intervals of 1 ms. However, the largest peak in the histogram appears at about 5 ms and not at the time corresponding to the inverse of 1000 pps as in the case for 250 pps. For 5000 pps (Fig. 10c), the histogram shows a single peak around 4 ms obtained with the temporal resolution used to construct the ISI histogram. The ISI histograms for 1000 and 5000 pps indicate that the neuron does not to generate a spike for each pulse in the pulse train. Hence, the largest peak in the distribution does not correspond to the period of the pulse train. Such transition between discrete ISI distributions at low pulse rates to more continuous distributions at higher pulse rates is consistent with data in Miller et al. (2008).

Figure 11 shows the VS of the neural response as a function of the stimulation pulse rate. The predictions (Fig. 11a) show values close to 1 for low pulse rates, decreasing to about 0.4 for 10,000 pps. This prediction is consistent with the data from Hartmann and Klinke (1990; downward pointing triangle for HK90) and Dynes and Delgutte (1992; upward pointing triangle for DD92) shown in Figure 11b. Thus, both data and predictions maintain a sustained synchronization to the stimulus frequencies even beyond 5000 pps.

Figure 12 shows the effect of the stimulus level on VS for pulse rates of 250, 1000, and 5000 pps. The dynamic range of the neural response depends on the stimulation pulse rate (as shown in Fig. 8). Therefore, the VS is shown as a function of the spikes/s instead of the stimulus level. For 250 pps (Fig. 12a) and 1000 pps (Fig. 12b), the VS approaches 1 with increasing spike rate. This indicates strong phase locking at higher levels for 250 and 1000 pps. For 5000 pps (Fig. 12c), the maximum value of the VS approached is about 0.7. The predictions represent the main trends observed in the data of Miller et al. (2008) shown in Figure 12d–f. However, for 5000 pps, the predictions deviate from the data in that they show a nonmonotonic effect of spike rate on the VS, whereas the data indicates no clear change in VS with increasing level (Fig. 12f).

**FIG. 13:**
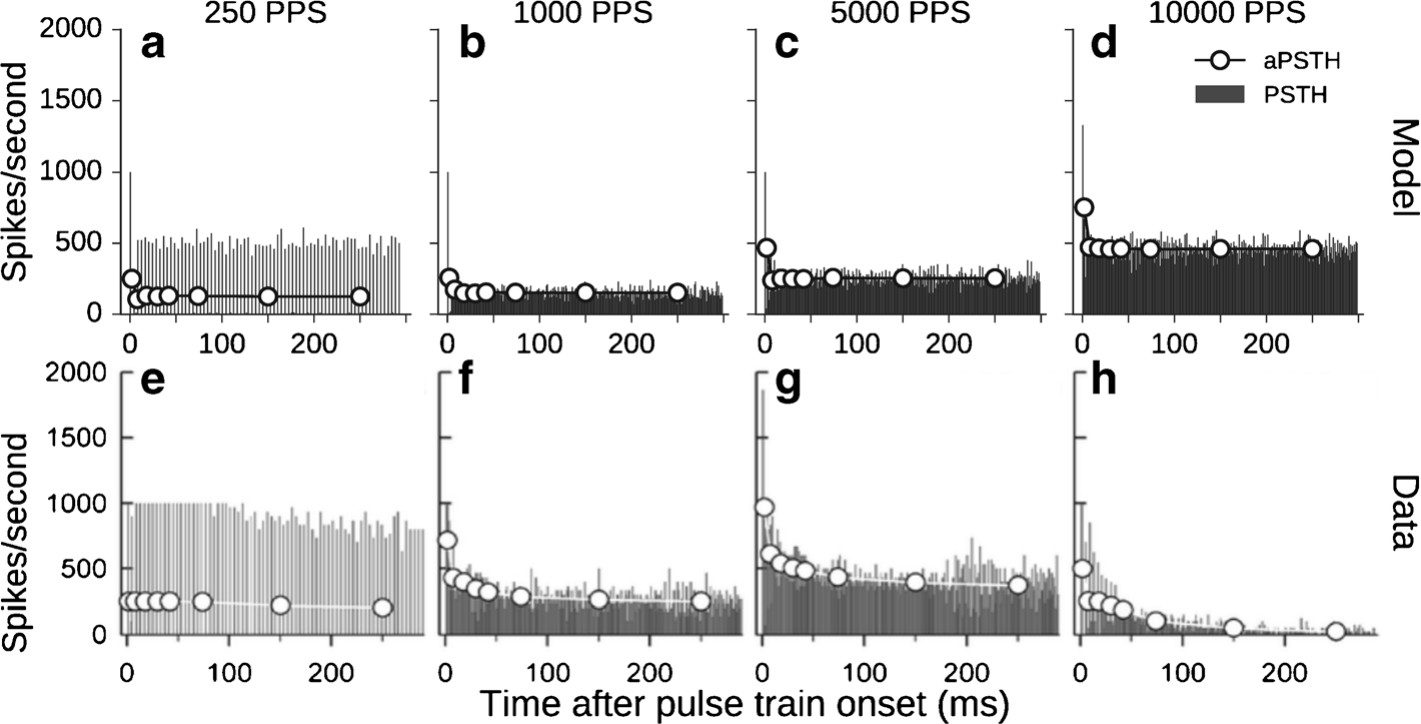
PSTH (*gray bars*) and aPSTH (*red circles*) constructed from the predicted responses for stimulation with pulse trains of 250 pps (**a**), 1000 pps (**b**), 5000 pps (**c**), and 10,000 pps (**d**) along with the corresponding data from Zhang et al. (2007) (**e**–**h**). The data have been reprinted with kind permission of the Journal of the Association for Research in Otolaryngology, Springer Science + Business Media: Fig. 2 from Miller et al. (2008), © Association for Research in Otolaryngology 2007.

Figure 13 shows the changes in spike rate over time in terms of the PSTH and aPSTH obtained from responses to stimulation rates of 250 pps (Fig. 13a), 1000 pps (Fig. 13b), 5000 pps (Fig. 13c), and 10,000 pps (Fig. 13d). The top row of Figure 13 shows PSTH (gray bars) constructed from the model responses. For 250 pps (Fig. 13a), the PSTH shows spikes occurring in response to each pulse of the pulse train. For higher pulse rates, a strong onset response is followed by a decrease in the spike rate. The decrease of the spike rate relative to the onset is larger for 1000 pps (Fig. 13b) than for 5000 (Fig. 13c) and 10,000 pps (Fig. 13d). This effect of pulse rate on the changes in response over time can also be observed in aPSTH which is indicated by the red circles. The first point of the aPSTH, which corresponds to the spike rate within the time period of the 0 to 4 ms from the stimulus onset, is always larger than the remaining points. This represents the onset response. The aPSTH show that the onset response becomes stronger with increasing pulse rate (Fig. 13a–d). After the onset response, there is a substantial reduction of the response that is observed, as is reflected in the aPSTH. The predictions follow the trends observed in the data of Zhang et al. (2007) for the pulse rates of 250, 1000, and 5000 pps, indicated in the bottom row in Figure 13e–g. However, the data for 10,000 pps (Fig. 13h) show that the response exhibits a large onset response followed by a strongly reduced spiking of the ANFs (Zhang et al. 2007). Such a “shutdown” of the neural activity following the strong onset response is accounted for by the model (Fig. 13d).

## **DISCUSSION**

### Summary of Main Findings

In this study, a computational model of the ANF responses to electrical stimulation was presented. The structure of the model was motivated by the experimental observation that both anodic and cathodic charges can generate a spike, whereby the site of spike generation differs for the two polarities. The model assumes two independent point neurons which mimic two sites of spike generation, namely the peripheral and the central axons of the ANF. The differences in sensitivity for anodic and cathodic charges was introduced in the model through the parameters derived from the rheobase and the chronaxie for monophasic pulses, using a procedure which describes the strength-duration function as a linear function instead of the traditionally considered exponential function. The model was evaluated in stimulus conditions with single pulses of various pulse shapes as well as with paired pulses and pulse trains. The results demonstrate that the charge integration properties of the neural membrane derived from responses to monophasic pulses can characterize the responsiveness of the neuron to electrical stimulation sufficiently well to account for the stimulation with biphasic pulses and pulses of alternative shapes.

The dynamic response properties of the ANF responses were modeled by two feedback current loops, describing the subthreshold and the suprathreshold currents which continuously modify the membrane voltage with respective time constants ($$ {\tau}_{\begin{array}{c}sub\\ {}\ \end{array}} $$ and $$ {\tau}_{\begin{array}{c}\mathrm{supra}\\ {}\ \end{array}} $$). The values of these adaptation time constants were adjusted to match the data obtained with paired pulse stimulation and were evaluated in conditions with pulse trains of various pulse rates and levels. The results demonstrated that this model can correctly predict the trends in the response statistics, such as the Fano factor and the VS. The parameters of the feedback current loops that were derived to match the data for paired pulse could, at least partly, be successfully applied to more dynamic stimuli such as pulse trains.

### Relation to Existing Models

Multiple phenomenological models of the ANF responses to electrical stimulation have been proposed (Bruce et al. 1999a, 1999b; Miller et al. 1999; Rubinstein et al. 2001; Litvak et al. 2003; Nourski et al. 2006; Macherey et al. 2007; Fredelake and Hohmann 2012; Goldwyn et al. 2012; Morse et al. 2015; Horne et al. 2016). These models do not consider multiple sites of spike generation and their effect on spike time statistics, and hence cannot be generalized to assess different CI stimulation strategies (Joshi et al. 2014). In contrast, the model presented in this study considers two sites of spike generation along the ANF as well as differences in their sensitivity to anodic and cathodic charges and is shown to account for the effect of different pulse shapes on the ANF response statistics.

The existing models also fail to predict the effects of subthreshold and suprathreshold stimuli on the responses to the following stimulation, which strongly affect the temporal responses of the ANF responses (Boulet et al. 2016). Only Goldwyn et al. (2012) used the summation threshold time constants to model facilitation, enabling their model to predict temporal integration effects across pulse trains. Nevertheless, their model did not include any effects of accommodation and therefore cannot account for accommodation observed in spike responses at different pulse rates. In the model proposed in the present study, the response properties of facilitation as well as the accommodation are accounted by the combination of the passive membrane filtering and the inclusion of a single subthreshold adaptive feedback loop.

The existing models simulate the stochasticity in the ANF responses using the stochastic threshold framework proposed by Bruce et al. (1999). In that framework, a spiking threshold is assumed to be random with a Gaussian distribution identical to that underlying the FE function and is approximated by white noise. In contrast, in the present study, the stochasticity is simulated as fluctuations in the membrane voltage using spectrally shaped noise (1/*f* ^*α*^) instead of the white noise. The use of 1/*f* ^*α*^ shaped noise produces physiologically realistic fluctuations of the membrane voltage (Verveen and Derksen 1965) and has also implications for the temporal response properties of the model. The 1/*f* ^*α*^ shaped spectrum of the noise enhances the coding of the lower frequencies in the ANF responses and results in correlated activity due to low frequency oscillations in the membrane across the multiple fibers (Pozzorini et al. 2013).

Furthermore, the existing models simulate the difference in thresholds for monophasic and biphasic pulses using a parameter called the activation time (Rubinstein et al. 2001). The higher thresholds observed for biphasic pulses have been attributed to the inhibitory properties of the second phase (van den Honert and Mortimer 1979). It has been proposed that there exists a time delay, the activation time, between the point in time when the neural membrane voltage has crossed a threshold for spiking and when the actual spike occurs; if an inhibitory charge occurs during this delay, the initiated spike can be canceled. Rubinstein et al. (2001) showed that the inclusion of a constant activation time delay in a simple linear integrate-to-threshold model can account for the threshold differences between monophasic and biphasic pulses. Such a delay has also been considered in recent models, such as the leaky integrate-and-fire model proposed by Horne et al. (2016). In contrast to that model, the model presented here does not require such activation time to account for the differences in thresholds between the monophasic and biphasic pulses, since the model does not indicate a spike when it crosses the threshold voltage. Instead, the membrane voltage starts to grow exponentially after it has crossed the threshold voltage. If enough inhibitory stimulation occurs during the exponential upswing of the membrane voltage, the spike is canceled. This inherent feature in the exponential integrate-and-fire neuron provides a biophysically relevant alternative to the use of an activation time.

Finally, it has been suggested that spike latency differences between the peripheral and the central axons occur due to a somatic delay. Accordingly, several models have assumed that spikes generated on the peripheral axon must pass through the soma where they are delayed due to a high capacitance of the soma (Rattay et al. 2001, 2013). However, the role of the soma in spike generation or spike conduction has not yet been confirmed experimentally. In fact, some biophysical models that do *not* include the soma and simulate the ANF as a uniform cable have also been successful in capturing various dynamics of the ANF responses (e.g., Imennov and Rubinstein 2009). The model presented in this study was also able to account for the differences in spike latencies between spikes generated at the peripheral and the central axons *without* assuming the passive compartment of the soma to add a delay to the spikes generated at the peripheral axons. Instead, latency difference was achieved by employing different values of the term ∆*T* in Eq. (2) between the two axons. Biophysically, ∆*T* has been related to the sharpness of the voltage-gated Na^+^ channel activation in the neural membrane of the ANF (Rutherford et al. 2012). Hence, the difference in ∆*T* between the two axons of the model may reflect the differences in dynamics of the voltage-gated Na^+^ ion channels in the peripheral and the central axons of the ANF. Indeed, significant differences in the distributions of voltage-gated ion channels along the ANFs have been reported (for review see Davis and Crozier 2016), but their roles in spike generation or conduction along the ANF are yet to be explored (Negm and Bruce, 2014; Boulet et al., 2016). Overall, the results of the present study suggest that while the two sites of spike generation are necessary to account for the differences in spike latencies, modeling the passive soma may not be necessary to capture the latency differences between the peripheral and the central axons.

### Limitations of the Model

The proposed model captures the essential features of the spike generation without modeling the biophysical details regarding the mechanism of the action potential generation. While many phenomena are captured reasonably well, it is challenging to attribute the model parameters to biophysical elements, such as distributions of various voltage-gated ion channels. The model components are physiologically inspired. Nevertheless, a direct biophysical interpretation of the model parameters to the detailed ion channel dynamics is not possible. The insights gained from this approach will, however, be useful for more detailed modeling approaches to identify the corresponding biophysical elements.

The model was shown to correctly capture the spike latency differences between the two sites of spike generation as they are excited by different polarities. Although the model predicts correct trends of spike latency differences, the absolute spike latencies reported in Miller et al. (1999) were about 200 μs longer than those predicted by the model (Fig. 3e). This might be due to the recording site in the experiment: in the physiological experiments, the ANF spikes were recorded at the root of the AN, where the ANFs make innervations with the cochlear nucleus. Consequently, the spike latencies measured at that location also include the duration it takes for the spike to travel along the ANF. Although the model presented here aims to mimic the responsiveness of both the peripheral and central nodes of the ANFs, it does not explicitly model the entire ANF through which a spike must travel. Since the offset is constant, the addition of a constant time delay of approximately 200 μs could be assumed to account for a complete travel time measured at the root of the ANF.

Although the model considers two sites of excitation, these point neurons are considered to be located in parallel rather than in series as they would be in the ANF. Since the two axons of the model independently integrate the stimulus charge, there is no interaction of the membrane voltage between the two axons. Instead, the interaction between the two axons is solely represented through the OR gate. Because of this architecture, the model only predicts the orthodromic spikes, i.e., the spikes traveling in afferent direction along the ANF. However, spikes generated along more central sites along the ANF can travel in efferent direction, known as the antidromic potential. Such an effect of the antidromic potential can affect the ECAP recordings (Miller et al. 2004) but has not been considered in the current framework. A development of alternative strategies to describe the interaction between the axons of the ANF is required to model an effect of antidromic potential on the ECAP responses.

While the model is mostly successful in predicting the effect of pulse rate on neural responses, the predictions of the temporal response properties deviate from the data at very high pulse rates, e.g., 10,000 pps. Measured ANF responses to high pulse rate stimulation have been characterized as dynamically unstable pattern, showing a strong onset followed by almost a shutdown of the neural responses (O’Gorman et al. 2009, 2010). The model, in its current form, is unable to capture such effect.

The parameters of the model were chosen to account for single fiber responses and to describe the trends in the data. It does not account for the observed variability in the data. However, due to its computationally efficient integrate-and-fire-type structure, the model can easily be extended to represent a population of neurons by considering repeated simulations of the model for a desired number of neurons. This is an advantage over complex biophysical models, which are computationally expensive and hence limit the simulations to fewer neurons. In combination with a current spread model, the presented model can also be used to investigate individual factors leading to the variability in the predictions. Such an approach would be useful to study the effect of various parameters such as the spiking threshold (e.g., van den Honert and Stypulkowski 1984; Miller et al. 1999) and refractoriness (e.g., Miller et al. 2001a) on population responses to dynamic stimuli, such as those reported by Matsuoka et al. (2000).

Finally, the model was developed based on responses recorded mainly from the ANFs of acutely deafened cats implanted with CI. It has been suggested that differences in sensitivity to anodic and cathodic currents may be dependent on the species under investigation. For example, recordings of the ANFs from guinea pigs show no significant differences between the thresholds for monophasic anodic and cathodic pulses. In contrast, in cats, the thresholds for anodic pulses are higher than for cathodic pulses (Miller et al. 1998, 1999) while in humans, the ANFs appear to be more sensitive to anodic than to cathodic pulses (Macherey et al. 2008). Despite the differences in sensitivity to anodic and cathodic currents, the differences in spike times between the two currents are consistent across species. Although the model presented here has been parameterized using the data for cats, it does not make explicit assumptions about differences in sensitivity to pulses of opposite currents. Hence, the model sensitivity to the current pulses will need to be modified by changing the membrane characteristics of the axons (and not the model structure), in order to account for the species specific differences in sensitivity.

## **CONCLUSION**

A computational model of ANF responses to electrical stimulation was presented. The model was inspired by the observation that charges of either polarity can generate a spike, but with different spike latencies. Based on the spike latency data for electrical stimulation with monophasic pulses of cat ANF, it was assumed here that the site of spike generation for monophasic anodic pulses is closer to the brainstem (central axon) than the site of spike generation for a monophasic, cathodic pulse (peripheral axon). The model was parametrized using limited data for monophasic stimulation, and model responses were compared to available data, mainly from electrically stimulated ANFs in cats. The model was shown to correctly account for stimulation with alternative pulse shapes as well as with dynamic stimuli such as pulse trains of different pulse rates and stimulus levels. Overall, the proposed model framework can serve as a useful tool to explore the temporal coding in the electrically stimulated ANF in response to various CI stimuli and to quantify potential benefits to the CI listeners.
